# Supplementary material for: A high-resolution mRNA expression time course of embryonic development in zebrafish
Source: eLife. 2017 Nov 16;6:e30860. doi: 10.7554/eLife.30860 (PMC5690287; doi:10.7554/eLife.30860)
Supplement: Supplementary file 6. [file elife-30860-supp6.zip › biolayout-clusters-files/Cluster168-genes.html]

Cluster168


# Cluster168: Genes

| Ensembl ID | Gene Name | Chr | Start | End | Biotype |
| --- | --- | --- | --- | --- | --- |
| ENSDARG00000103665 | BRPF1 | 8 | 54034356 | 54053810 | protein\_coding |
| ENSDARG00000094018 | ENSDARG00000094018 | 5 | 37381244 | 37397635 | protein\_coding |
| ENSDARG00000028431 | adnp2b | 19 | 21201401 | 21208150 | protein\_coding |
| ENSDARG00000037091 | asah1a | 14 | 29932577 | 29938094 | protein\_coding |
| ENSDARG00000069085 | ints2 | 10 | 28090748 | 28142374 | protein\_coding |
| ENSDARG00000041503 | med4 | 9 | 25509811 | 25517658 | protein\_coding |
| ENSDARG00000016181 | trim33 | 8 | 10918763 | 10967034 | protein\_coding |
| ENSDARG00000069114 | unc93b1 | 1 | 43694673 | 43716922 | protein\_coding |
